# Preface

**Published:** 2006

*What Medicine Means To Me is* a first in many ways for *MSM.* This is the first Theme Monograph. It is also the first Monograph which has contributions from the Asian, Australian, American and African continents.

**Donelson Dulany**, Professor of Psychology Emeritus at the University of Illinois and Editor, *American Journal of Psychology,* writes on, ′What Psychology Means To Me’ (p36 ).

**Sunil K. Pandya,** who writes on ‘Where is Medical Practice In India Heading?’ (p50) and also on ‘Turning Points In My Medical Career’ (p154 ), is a Neurosurgeon and from the Dept of Neurosurgery, Jaslok Hospital And Research Center, Mumbai, India. He is also Editor Emeritus, *Indian Journal Of Medical Ethics,* and on the International Advisory Board of *MSM.*

**Shaukat Ali Jawaid,** who writes on ‘What Medicine And Medical Journal Editing Mean To Me’ (p62 ), is Chief Editor, *Pulse International,* and Managing Editor, *Pakistan Journal of Medical Sciences,* Karachi, Pakistan.

**Adamson S. Muula,** who writes on ‘Medicine and Money: Friends Or Foe?’ (p78 ) is from the Department of Community Health, University of Malawi, College of Medicine, Malawi and Department of Epidemiology, University of North Carolina at Chapel Hill, United States.

**Vance W. Berger,** from the National Cancer Institute, Bethesda, MD, USA, and **J. Rosser Matthews,** from the Virginia Commonwealth University, write on, ‘What Does Biostatistics Mean to Us’ (p89).

**Morten Hesse,** who writes on ‘What Does Addiction Mean to Me’ is from Aarhus University, Centre for Alcohol and Drug Research, Købmagergade 26E, DK-1150 Copenhagen (p104).

**S.C. Panda,** who writes on ‘Medicine: Science or Art?’ is Assistant Professor, Department of Community Medicine, V.S.S. Medical College, Burla, Orissa, and Editor, *Journal of Community Medicine* (p127).

**Roy Sugarman,** Acting Director of Psychology at Royal Rehabilitation Centre in Sydney, and Conjoint Senior Lecturer in Psychiatry at the University of New South Wales, writes on, ‘What Psychology Means to Me’ (p139).

**J. K. Trivedi,** Professor, Department of Psychiatry, and **Dishanter Goel,** Junior Resident, both from the King George's Medical University, Lucknow, India, write on, ‘What Psychiatry Means To Us’ (p166). JKT has also been Past Editor, *Indian Journal of Psychiatry,* Past President, Indian Psychiatric Society, and is a Member of the International Advisory Board of *MSM.*

**S. Malhotra** and **N. Shafiq,** from the Department of Pharmacology, Postgraduate Institute of Medical Education and Research, Chandigarh, India, write on, ‘What Clinical Pharmacology Means to Us’ (p184).

**Ajai R. Singh,** Editor and **Shakuntala A. Singh,** Deputy Editor, *MSM,* write Editorials on ‘To Cure Sometimes, To Comfort Always, To Hurt The Least, To Harm Never’ (p8), ‘Psychiatrists And Clinical Psychologists’ (p10), and ‘What Is A Good Editorial?’ (p14), besides writing for the Looking Mirror, ‘A look at *CMAJ:* A Misty Image Indeed’ (p21). **Ajai R. Singh** also writes Musings (p18), and in MSM Poems (p208).

**Avinash De Sousa,** Consulting Psychiatrist, Mumbai, writes an Obituary for his mother in, ‘Dr. Mrs. Dhanalakshmi De Sousa.1938–2005.A Tribute’ (p211).

**K.P. Dave,** retired Head, Dept. of Psychiatry, L.T.M.G. Medical College and Hospital, Sion, Mumbai, India, also writes an obituary on ‘Dr. Mrs. Dhanalakshmi De Sousa (p213).

The writings present a rich and varied fare for the serious reader.

